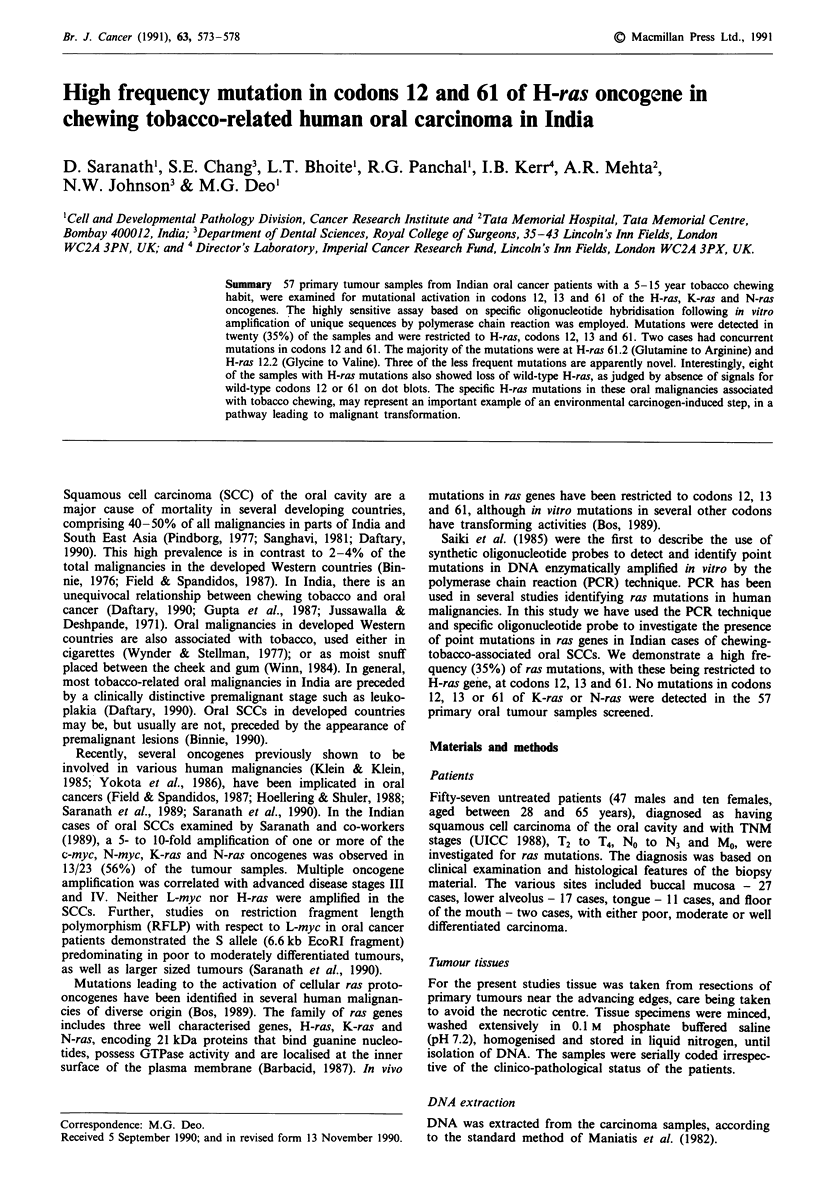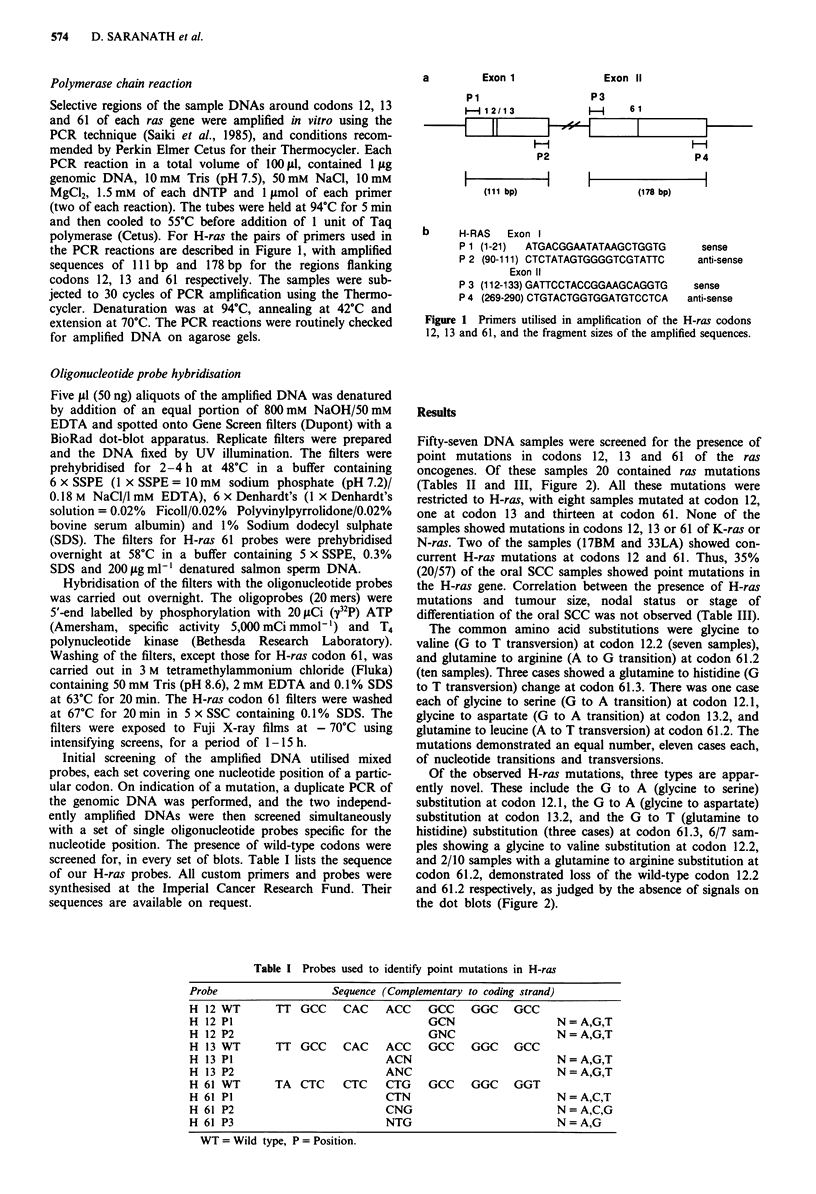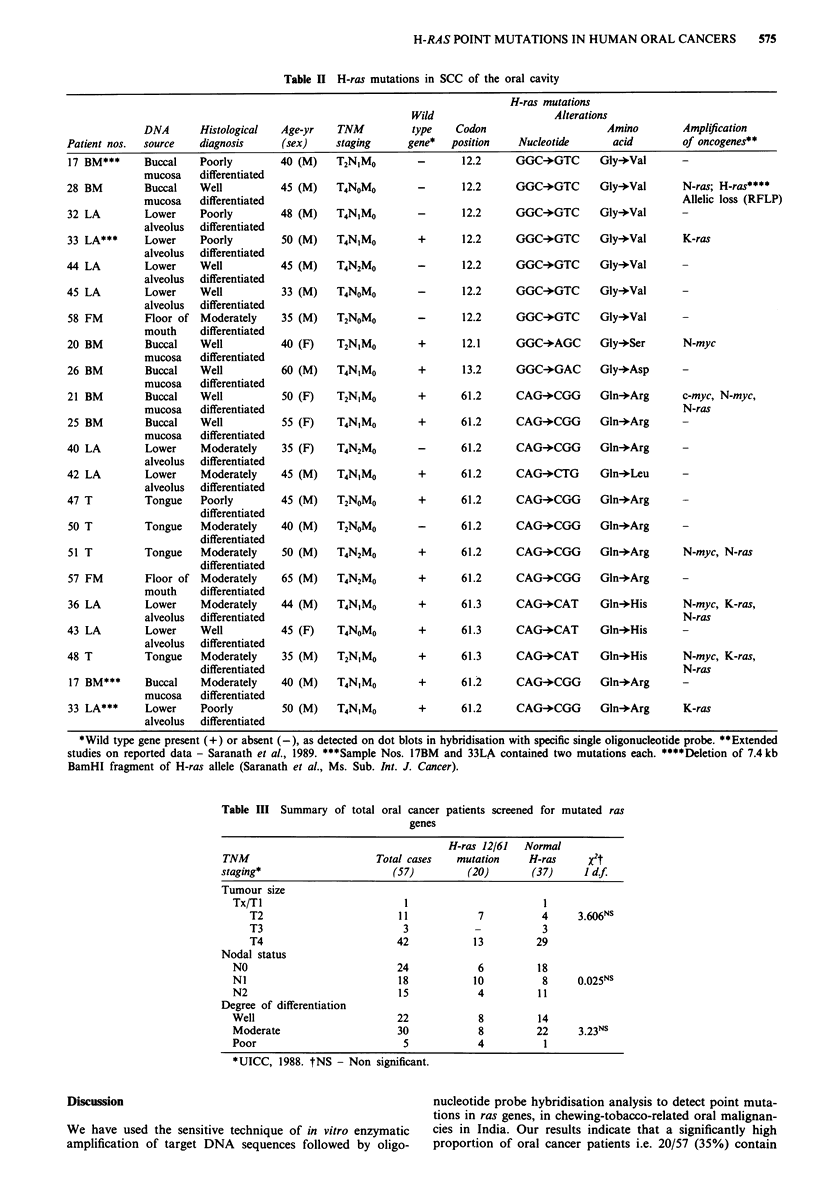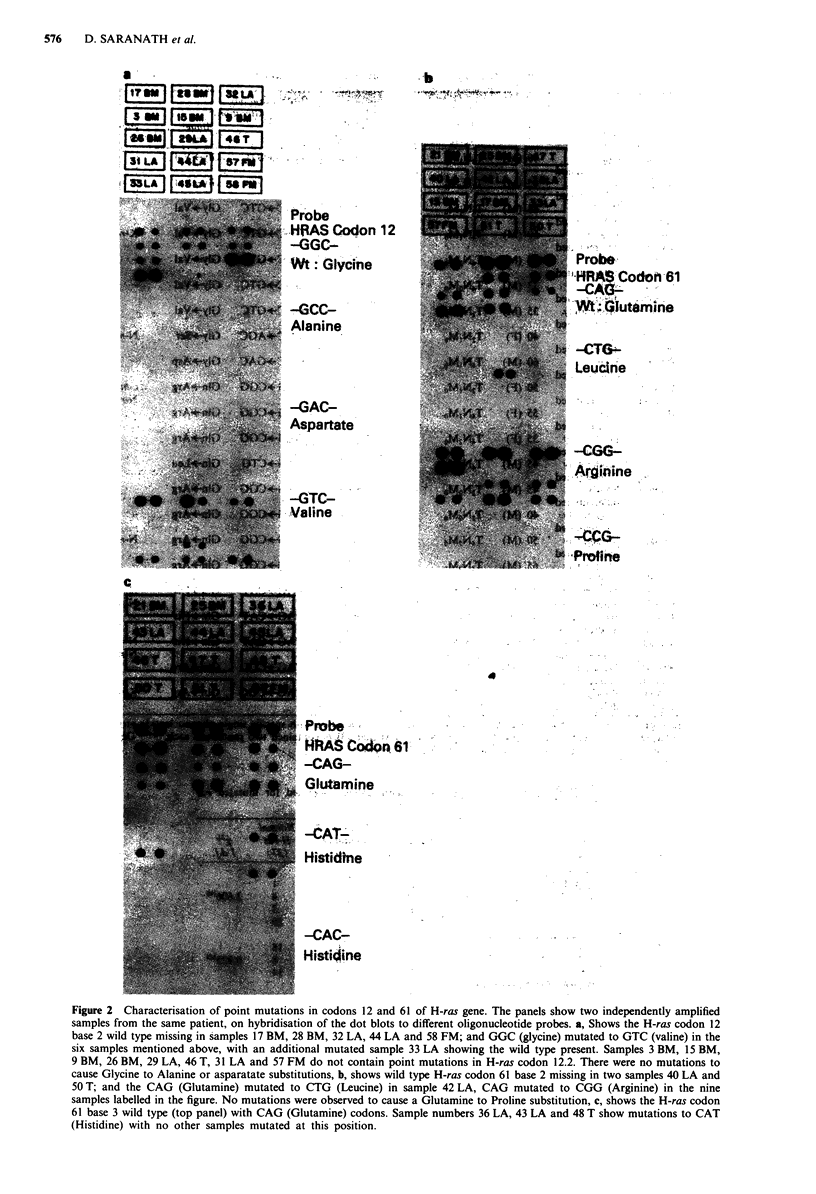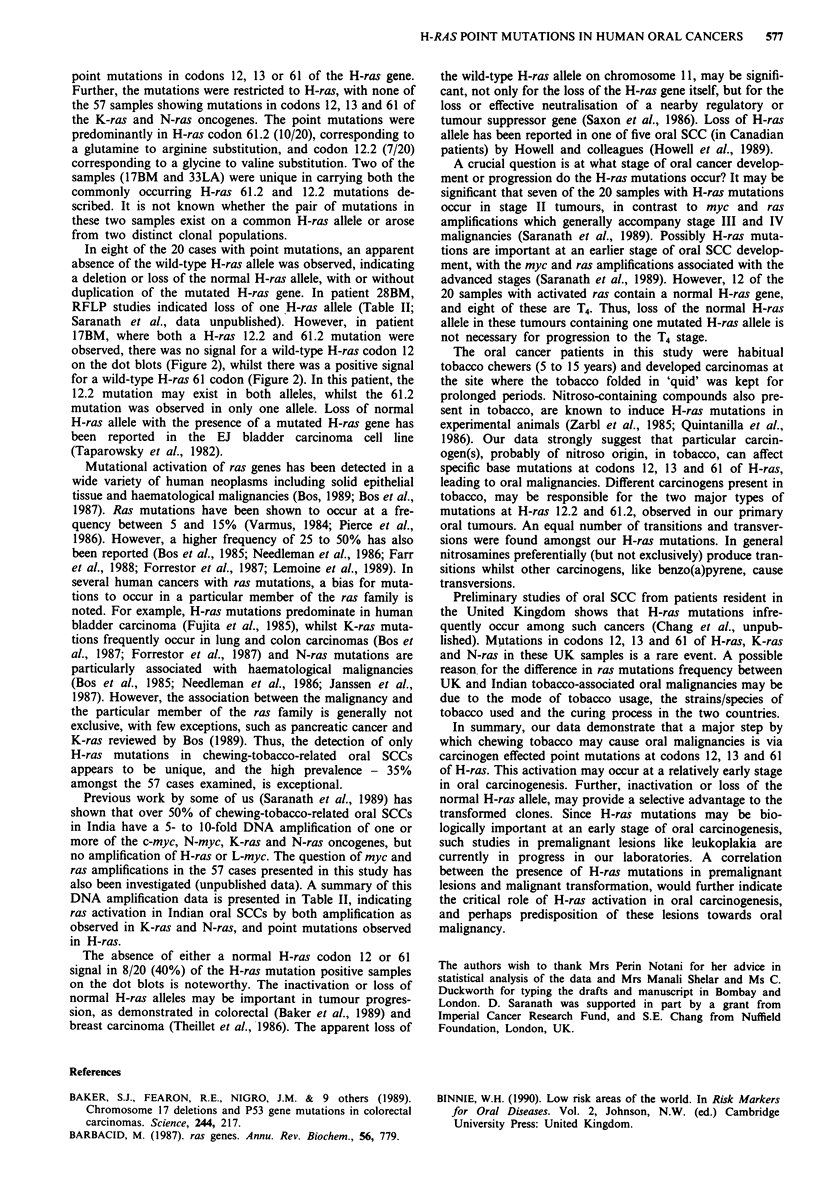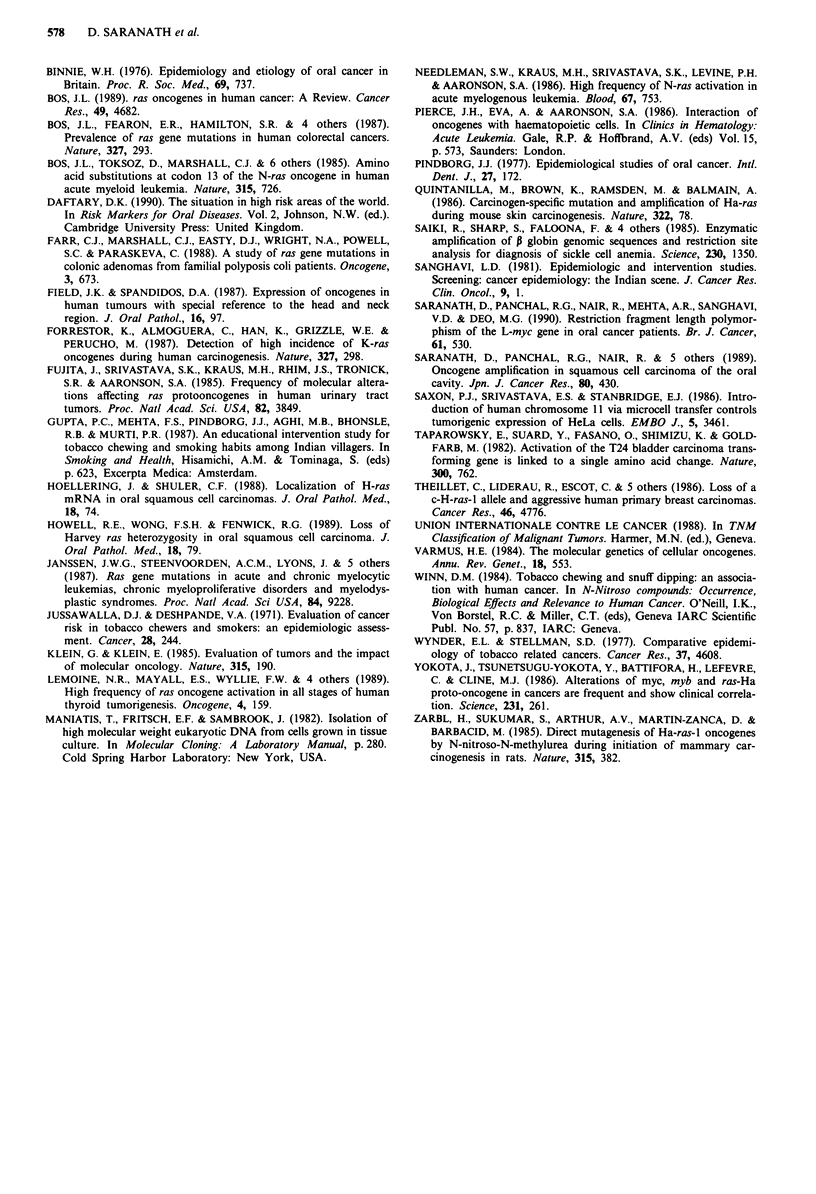# High frequency mutation in codons 12 and 61 of H-ras oncogene in chewing tobacco-related human oral carcinoma in India.

**DOI:** 10.1038/bjc.1991.133

**Published:** 1991-04

**Authors:** D. Saranath, S. E. Chang, L. T. Bhoite, R. G. Panchal, I. B. Kerr, A. R. Mehta, N. W. Johnson, M. G. Deo

**Affiliations:** Cell and Developmental Pathology Division, Cancer Research Institute, Bombay, India.

## Abstract

**Images:**


Br. J. Cancer (1991), 63, 573-578 © Macmillan Press Ltd., 1991

### High frequency mutation in codons 12 and 61 of H-*ras* oncogene in chewing tobacco-related human oral carcinoma in India

D. Saranath[1], S.E. Chang[3], L.T. Bhoite[1], R.G. Panchal[1], I.B. Kerr[4], A.R. Mehta[2], N.W. Johnson[3] & M.G. Deo[1]

[1]*Cell and Developmental Pathology Division, Cancer Research Institute and* [2]*Tata Memorial Hospital, Tata Memorial Centre, Bombay 400012, India;* [3]*Department of Dental Sciences, Royal College of Surgeons, 35-43 Lincoln's Inn Fields, London WC2A 3PN, UK; and* [4] *Director's Laboratory, Imperial Cancer Research Fund, Lincoln's Inn Fields, London WC2A 3PX, UK.*

**Summary** 57 primary tumour samples from Indian oral cancer patients with a 5-15 year tobacco chewing habit, were examined for mutational activation in codons 12, 13 and 61 of the H-*ras*, K-*ras* and N-*ras* oncogenes. The highly sensitive assay based on specific oligonucleotide hybridisation following *in vitro* amplification of unique sequences by polymerase chain reaction was employed. Mutations were detected in twenty (35%) of the samples and were restricted to H-*ras*, codons 12, 13 and 61. Two cases had concurrent mutations in codons 12 and 61. The majority of the mutations were at H-*ras* 61.2 (Glutamine to Arginine) and H-*ras* 12.2 (Glycine to Valine). Three of the less frequent mutations are apparently novel. Interestingly, eight of the samples with H-*ras* mutations also showed loss of wild-type H-*ras*, as judged by absence of signals for wild-type codons 12 or 61 on dot blots. The specific H-*ras* mutations in these oral malignancies associated with tobacco chewing, may represent an important example of an environmental carcinogen-induced step, in a pathway leading to malignant transformation.

Squamous cell carcinoma (SCC) of the oral cavity are a major cause of mortality in several developing countries, comprising 40-50% of all malignancies in parts of India and South East Asia (Pindborg, 1977; Sanghavi, 1981; Daftary, 1990). This high prevalence is in contrast to 2-4% of the total malignancies in the developed Western countries (Binnie, 1976; Field & Spandidos, 1987). In India, there is an unequivocal relationship between chewing tobacco and oral cancer (Daftary, 1990; Gupta *et al.*, 1987; Jussawalla & Deshpande, 1971). Oral malignancies in developed Western countries are also associated with tobacco, used either in cigarettes (Wynder & Stellman, 1977); or as moist snuff placed between the cheek and gum (Winn, 1984). In general, most tobacco-related oral malignancies in India are preceded by a clinically distinctive premalignant stage such as leukoplakia (Daftary, 1990). Oral SCCs in developed countries may be, but usually are not, preceded by the appearance of premalignant lesions (Binnie, 1990).

Recently, several oncogenes previously shown to be involved in various human malignancies (Klein & Klein, 1985; Yokota *et al.*, 1986), have been implicated in oral cancers (Field & Spandidos, 1987; Hoellering & Shuler, 1988; Saranath *et al.*, 1989; Saranath *et al.*, 1990). In the Indian cases of oral SCCs examined by Saranath and co-workers (1989), a 5- to 10-fold amplification of one or more of the c-*myc*, N-*myc*, K-*ras* and N-*ras* oncogenes was observed in 13/23 (56%) of the tumour samples. Multiple oncogene amplification was correlated with advanced disease stages III and IV. Neither L-*myc* nor H-*ras* were amplified in the SCCs. Further, studies on restriction fragment length polymorphism (RFLP) with respect to L-*myc* in oral cancer patients demonstrated the S allele (6.6 kb EcoRI fragment) predominating in poor to moderately differentiated tumours, as well as larger sized tumours (Saranath *et al.*, 1990).

Mutations leading to the activation of cellular *ras* proto-oncogenes have been identified in several human malignancies of diverse origin (Bos, 1989). The family of *ras* genes includes three well characterised genes, H-*ras*, K-*ras* and N-*ras*, encoding 21 kDa proteins that bind guanine nucleotides, possess GTPase activity and are localised at the inner surface of the plasma membrane (Barbacid, 1987). *In vivo* mutations in *ras* genes have been restricted to codons 12, 13 and 61, although *in vitro* mutations in several other codons have transforming activities (Bos, 1989).

Saiki *et al.* (1985) were the first to describe the use of synthetic oligonucleotide probes to detect and identify point mutations in DNA enzymatically amplified *in vitro* by the polymerase chain reaction (PCR) technique. PCR has been used in several studies identifying *ras* mutations in human malignancies. In this study we have used the PCR technique and specific oligonucleotide probe to investigate the presence of point mutations in *ras* genes in Indian cases of chewing-tobacco-associated oral SCCs. We demonstrate a high frequency (35%) of *ras* mutations, with these being restricted to H-*ras* gene, at codons 12, 13 and 61. No mutations in codons 12, 13 or 61 of K-*ras* or N-*ras* were detected in the 57 primary oral tumour samples screened.

### Materials and methods

### *Patients*

Fifty-seven untreated patients (47 males and ten females, aged between 28 and 65 years), diagnosed as having squamous cell carcinoma of the oral cavity and with TNM stages (UICC 1988), $T_2$ to $T_4$, $N_0$ to $N_3$ and $M_0$, were investigated for *ras* mutations. The diagnosis was based on clinical examination and histological features of the biopsy material. The various sites included buccal mucosa - 27 cases, lower alveolus - 17 cases, tongue - 11 cases, and floor of the mouth - two cases, with either poor, moderate or well differentiated carcinoma.

### *Tumour tissues*

For the present studies tissue was taken from resections of primary tumours near the advancing edges, care being taken to avoid the necrotic centre. Tissue specimens were minced, washed extensively in 0.1 M phosphate buffered saline (pH 7.2), homogenised and stored in liquid nitrogen, until isolation of DNA. The samples were serially coded irrespective of the clinico-pathological status of the patients.

### *DNA extraction*

DNA was extracted from the carcinoma samples, according to the standard method of Maniatis *et al.* (1982).

Correspondence: M.G. Deo.
Received 5 September 1990; and in revised form 13 November 1990.

*Polymerase chain reaction*

Selective regions of the sample DNAs around codons 12, 13 and 61 of each *ras* gene were amplified *in vitro* using the PCR technique (Saiki *et al.*, 1985), and conditions recommended by Perkin Elmer Cetus for their Thermocycler. Each PCR reaction in a total volume of 100 µl, contained 1 µg genomic DNA, 10 mM Tris (pH 7.5), 50 mM NaCl, 10 mM $MgCl_2$, 1.5 mM of each dNTP and 1 µmol of each primer (two of each reaction). The tubes were held at 94°C for 5 min and then cooled to 55°C before addition of 1 unit of Taq polymerase (Cetus). For H-*ras* the pairs of primers used in the PCR reactions are described in Figure 1, with amplified sequences of 111 bp and 178 bp for the regions flanking codons 12, 13 and 61 respectively. The samples were subjected to 30 cycles of PCR amplification using the Thermocycler. Denaturation was at 94°C, annealing at 42°C and extension at 70°C. The PCR reactions were routinely checked for amplified DNA on agarose gels.

*Oligonucleotide probe hybridisation*

Five µl (50 ng) aliquots of the amplified DNA was denatured by addition of an equal portion of 800 mM NaOH/50 mM EDTA and spotted onto Gene Screen filters (Dupont) with a BioRad dot-blot apparatus. Replicate filters were prepared and the DNA fixed by UV illumination. The filters were prehybridised for 2–4 h at 48°C in a buffer containing 6 × SSPE (1 × SSPE = 10 mM sodium phosphate (pH 7.2)/ 0.18 M NaCl/1 mM EDTA), 6 × Denhardt's (1 × Denhardt's solution = 0.02% Ficoll/0.02% Polyvinylpyrrolidone/0.02% bovine serum albumin) and 1% Sodium dodecyl sulphate (SDS). The filters for H-*ras* 61 probes were prehybridised overnight at 58°C in a buffer containing 5 × SSPE, 0.3% SDS and 200 µg $ml^{-1}$ denatured salmon sperm DNA.

Hybridisation of the filters with the oligonucleotide probes was carried out overnight. The oligoprobes (20 mers) were 5′-end labelled by phosphorylation with 20 µCi ($\gamma^{32}P$) ATP (Amersham, specific activity 5,000 mCi $mmol^{-1}$) and $T_4$ polynucleotide kinase (Bethesda Research Laboratory). Washing of the filters, except those for H-*ras* codon 61, was carried out in 3 M tetramethylammonium chloride (Fluka) containing 50 mM Tris (pH 8.6), 2 mM EDTA and 0.1% SDS at 63°C for 20 min. The H-*ras* codon 61 filters were washed at 67°C for 20 min in 5 × SSC containing 0.1% SDS. The filters were exposed to Fuji X-ray films at −70°C using intensifying screens, for a period of 1–15 h.

Initial screening of the amplified DNA utilised mixed probes, each set covering one nucleotide position of a particular codon. On indication of a mutation, a duplicate PCR of the genomic DNA was performed, and the two independently amplified DNAs were then screened simultaneously with a set of single oligonucleotide probes specific for the nucleotide position. The presence of wild-type codons were screened for, in every set of blots. Table I lists the sequence of our H-*ras* probes. All custom primers and probes were synthesised at the Imperial Cancer Research Fund. Their sequences are available on request.

a b H-RAS Exon I

P 1 (1-21) ATGACGGAATATAAGCTGGTG sense

P 2 (90-111) CTCTATAGTGGGGTCGTATTC anti-sense

Exon II

P 3 (112-133) GATTCCTACCGGAAGCAGGTG sense

P 4 (269-290) CTGTACTGGTGGATGTCCTCA anti-sense

**Figure 1** Primers utilised in amplification of the H-*ras* codons 12, 13 and 61, and the fragment sizes of the amplified sequences.

### Results

Fifty-seven DNA samples were screened for the presence of point mutations in codons 12, 13 and 61 of the *ras* oncogenes. Of these samples 20 contained *ras* mutations (Tables II and III, Figure 2). All these mutations were restricted to H-*ras*, with eight samples mutated at codon 12, one at codon 13 and thirteen at codon 61. None of the samples showed mutations in codons 12, 13 or 61 of K-*ras* or N-*ras*. Two of the samples (17BM and 33LA) showed concurrent H-*ras* mutations at codons 12 and 61. Thus, 35% (20/57) of the oral SCC samples showed point mutations in the H-*ras* gene. Correlation between the presence of H-*ras* mutations and tumour size, nodal status or stage of differentiation of the oral SCC was not observed (Table III).

The common amino acid substitutions were glycine to valine (G to T transversion) at codon 12.2 (seven samples), and glutamine to arginine (A to G transition) at codon 61.2 (ten samples). Three cases showed a glutamine to histidine (G to T transversion) change at codon 61.3. There was one case each of glycine to serine (G to A transition) at codon 12.1, glycine to aspartate (G to A transition) at codon 13.2, and glutamine to leucine (A to T transversion) at codon 61.2. The mutations demonstrated an equal number, eleven cases each, of nucleotide transitions and transversions.

Of the observed H-*ras* mutations, three types are apparently novel. These include the G to A (glycine to serine) substitution at codon 12.1, the G to A (glycine to aspartate) substitution at codon 13.2, and the G to T (glutamine to histidine) substitution (three cases) at codon 61.3, 6/7 samples showing a glycine to valine substitution at codon 12.2, and 2/10 samples with a glutamine to arginine substitution at codon 61.2, demonstrated loss of the wild-type codon 12.2 and 61.2 respectively, as judged by the absence of signals on the dot blots (Figure 2).

**Table I** Probes used to identify point mutations in H-*ras*

| Probe | Sequence (Complementary to coding strand) | | | | | | | |
|---|---|---|---|---|---|---|---|---|
| H 12 WT | TT GCC | CAC | ACC | GCC | GGC | GCC | | |
| H 12 P1 | | | | GCN | | | | N = A,G,T |
| H 12 P2 | | | | GNC | | | | N = A,G,T |
| H 13 WT | TT GCC | CAC | ACC | GCC | GGC | GCC | | |
| H 13 P1 | | | ACN | | | | | N = A,G,T |
| H 13 P2 | | | ANC | | | | | N = A,G,T |
| H 61 WT | TA CTC | CTC | CTG | GCC | GGC | GGT | | |
| H 61 P1 | | | CTN | | | | | N = A,C,T |
| H 61 P2 | | | CNG | | | | | N = A,C,G |
| H 61 P3 | | | NTG | | | | | N = A,G |

WT = Wild type, P = Position.

**Table II** H-*ras* mutations in SCC of the oral cavity

| Patient nos. | DNA source | Histological diagnosis | Age-yr (sex) | TNM staging | Wild type gene* | H-ras mutations: Codon position | Alterations: Nucleotide | Alterations: Amino acid | Amplification of oncogenes** |
|---|---|---|---|---|---|---|---|---|---|
| 17 BM*** | Buccal mucosa | Poorly differentiated | 40 (M) | $T_2N_1M_0$ | – | 12.2 | GGC→GTC | Gly→Val | – |
| 28 BM | Buccal mucosa | Well differentiated | 45 (M) | $T_4N_0M_0$ | – | 12.2 | GGC→GTC | Gly→Val | N-*ras*; H-*ras***** Allelic loss (RFLP) |
| 32 LA | Lower alveolus | Poorly differentiated | 48 (M) | $T_4N_1M_0$ | – | 12.2 | GGC→GTC | Gly→Val | – |
| 33 LA*** | Lower alveolus | Poorly differentiated | 50 (M) | $T_4N_1M_0$ | + | 12.2 | GGC→GTC | Gly→Val | K-*ras* |
| 44 LA | Lower alveolus | Well differentiated | 45 (M) | $T_4N_2M_0$ | – | 12.2 | GGC→GTC | Gly→Val | – |
| 45 LA | Lower alveolus | Well differentiated | 33 (M) | $T_4N_0M_0$ | – | 12.2 | GGC→GTC | Gly→Val | – |
| 58 FM | Floor of mouth | Moderately differentiated | 35 (M) | $T_2N_0M_0$ | – | 12.2 | GGC→GTC | Gly→Val | – |
| 20 BM | Buccal mucosa | Well differentiated | 40 (F) | $T_2N_1M_0$ | + | 12.1 | GGC→AGC | Gly→Ser | N-*myc* |
| 26 BM | Buccal mucosa | Well differentiated | 60 (M) | $T_4N_1M_0$ | + | 13.2 | GGC→GAC | Gly→Asp | – |
| 21 BM | Buccal mucosa | Well differentiated | 50 (F) | $T_2N_1M_0$ | + | 61.2 | CAG→CGG | Gln→Arg | c-*myc*, N-*myc*, N-*ras* |
| 25 BM | Buccal mucosa | Well differentiated | 55 (F) | $T_4N_1M_0$ | + | 61.2 | CAG→CGG | Gln→Arg | – |
| 40 LA | Lower alveolus | Moderately differentiated | 35 (F) | $T_4N_2M_0$ | – | 61.2 | CAG→CGG | Gln→Arg | – |
| 42 LA | Lower alveolus | Moderately differentiated | 45 (M) | $T_4N_1M_0$ | + | 61.2 | CAG→CTG | Gln→Leu | – |
| 47 T | Tongue | Poorly differentiated | 45 (M) | $T_2N_0M_0$ | + | 61.2 | CAG→CGG | Gln→Arg | – |
| 50 T | Tongue | Moderately differentiated | 40 (M) | $T_2N_0M_0$ | – | 61.2 | CAG→CGG | Gln→Arg | – |
| 51 T | Tongue | Moderately differentiated | 50 (M) | $T_4N_2M_0$ | + | 61.2 | CAG→CGG | Gln→Arg | N-*myc*, N-*ras* |
| 57 FM | Floor of mouth | Moderately differentiated | 65 (M) | $T_4N_2M_0$ | + | 61.2 | CAG→CGG | Gln→Arg | – |
| 36 LA | Lower alveolus | Moderately differentiated | 44 (M) | $T_4N_1M_0$ | + | 61.3 | CAG→CAT | Gln→His | N-*myc*, K-*ras*, N-*ras* |
| 43 LA | Lower alveolus | Well differentiated | 45 (F) | $T_4N_0M_0$ | + | 61.3 | CAG→CAT | Gln→His | – |
| 48 T | Tongue | Moderately differentiated | 35 (M) | $T_2N_1M_0$ | + | 61.3 | CAG→CAT | Gln→His | N-*myc*, K-*ras*, N-*ras* |
| 17 BM*** | Buccal mucosa | Moderately differentiated | 40 (M) | $T_4N_1M_0$ | + | 61.2 | CAG→CGG | Gln→Arg | – |
| 33 LA*** | Lower alveolus | Poorly differentiated | 50 (M) | $T_4N_1M_0$ | + | 61.2 | CAG→CGG | Gln→Arg | K-*ras* |

*Wild type gene present (+) or absent (–), as detected on dot blots in hybridisation with specific single oligonucleotide probe. **Extended studies on reported data – Saranath *et al.*, 1989. ***Sample Nos. 17BM and 33LA contained two mutations each. ****Deletion of 7.4 kb BamHI fragment of H-*ras* allele (Saranath *et al.*, Ms. Sub. *Int. J. Cancer*).

**Table III** Summary of total oral cancer patients screened for mutated *ras* genes

| TNM staging* | Total cases (57) | H-ras 12/61 mutation (20) | Normal H-ras (37) | $\chi^2$† 1 d.f. |
|---|---|---|---|---|
| Tumour size | | | | |
| Tx/T1 | 1 | | 1 | |
| T2 | 11 | 7 | 4 | $3.606^{NS}$ |
| T3 | 3 | – | 3 | |
| T4 | 42 | 13 | 29 | |
| Nodal status | | | | |
| N0 | 24 | 6 | 18 | |
| N1 | 18 | 10 | 8 | $0.025^{NS}$ |
| N2 | 15 | 4 | 11 | |
| Degree of differentiation | | | | |
| Well | 22 | 8 | 14 | |
| Moderate | 30 | 8 | 22 | $3.23^{NS}$ |
| Poor | 5 | 4 | 1 | |

*UICC, 1988. †NS – Non significant.

### Discussion

We have used the sensitive technique of *in vitro* enzymatic amplification of target DNA sequences followed by oligonucleotide probe hybridisation analysis to detect point mutations in *ras* genes, in chewing-tobacco-related oral malignancies in India. Our results indicate that a significantly high proportion of oral cancer patients i.e. 20/57 (35%) contain

**Figure 2** Characterisation of point mutations in codons 12 and 61 of H-*ras* gene. The panels show two independently amplified samples from the same patient, on hybridisation of the dot blots to different oligonucleotide probes. **a**, Shows the H-*ras* codon 12 base 2 wild type missing in samples 17 BM, 28 BM, 32 LA, 44 LA and 58 FM; and GGC (glycine) mutated to GTC (valine) in the six samples mentioned above, with an additional mutated sample 33 LA showing the wild type present. Samples 3 BM, 15 BM, 9 BM, 26 BM, 29 LA, 46 T, 31 LA and 57 FM do not contain point mutations in H-*ras* codon 12.2. There were no mutations to cause Glycine to Alanine or asparatate substitutions, **b**, shows wild type H-*ras* codon 61 base 2 missing in two samples 40 LA and 50 T; and the CAG (Glutamine) mutated to CTG (Leucine) in sample 42 LA, CAG mutated to CGG (Arginine) in the nine samples labelled in the figure. No mutations were observed to cause a Glutamine to Proline substitution, **c**, shows the H-*ras* codon 61 base 3 wild type (top panel) with CAG (Glutamine) codons. Sample numbers 36 LA, 43 LA and 48 T show mutations to CAT (Histidine) with no other samples mutated at this position.

point mutations in codons 12, 13 or 61 of the H-*ras* gene. Further, the mutations were restricted to H-*ras*, with none of the 57 samples showing mutations in codons 12, 13 and 61 of the K-*ras* and N-*ras* oncogenes. The point mutations were predominantly in H-*ras* codon 61.2 (10/20), corresponding to a glutamine to arginine substitution, and codon 12.2 (7/20) corresponding to a glycine to valine substitution. Two of the samples (17BM and 33LA) were unique in carrying both the commonly occurring H-*ras* 61.2 and 12.2 mutations described. It is not known whether the pair of mutations in these two samples exist on a common H-*ras* allele or arose from two distinct clonal populations.

In eight of the 20 cases with point mutations, an apparent absence of the wild-type H-*ras* allele was observed, indicating a deletion or loss of the normal H-*ras* allele, with or without duplication of the mutated H-*ras* gene. In patient 28BM, RFLP studies indicated loss of one H-*ras* allele (Table II; Saranath *et al.*, data unpublished). However, in patient 17BM, where both a H-*ras* 12.2 and 61.2 mutation were observed, there was no signal for a wild-type H-*ras* codon 12 on the dot blots (Figure 2), whilst there was a positive signal for a wild-type H-*ras* 61 codon (Figure 2). In this patient, the 12.2 mutation may exist in both alleles, whilst the 61.2 mutation was observed in only one allele. Loss of normal H-*ras* allele with the presence of a mutated H-*ras* gene has been reported in the EJ bladder carcinoma cell line (Taparowsky *et al.*, 1982).

Mutational activation of *ras* genes has been detected in a wide variety of human neoplasms including solid epithelial tissue and haematological malignancies (Bos, 1989; Bos *et al.*, 1987). *Ras* mutations have been shown to occur at a frequency between 5 and 15% (Varmus, 1984; Pierce *et al.*, 1986). However, a higher frequency of 25 to 50% has also been reported (Bos *et al.*, 1985; Needleman *et al.*, 1986; Farr *et al.*, 1988; Forrestor *et al.*, 1987; Lemoine *et al.*, 1989). In several human cancers with *ras* mutations, a bias for mutations to occur in a particular member of the *ras* family is noted. For example, H-*ras* mutations predominate in human bladder carcinoma (Fujita *et al.*, 1985), whilst K-*ras* mutations frequently occur in lung and colon carcinomas (Bos *et al.*, 1987; Forrestor *et al.*, 1987) and N-*ras* mutations are particularly associated with haematological malignancies (Bos *et al.*, 1985; Needleman *et al.*, 1986; Janssen *et al.*, 1987). However, the association between the malignancy and the particular member of the *ras* family is generally not exclusive, with few exceptions, such as pancreatic cancer and K-*ras* reviewed by Bos (1989). Thus, the detection of only H-*ras* mutations in chewing-tobacco-related oral SCCs appears to be unique, and the high prevalence – 35% amongst the 57 cases examined, is exceptional.

Previous work by some of us (Saranath *et al.*, 1989) has shown that over 50% of chewing-tobacco-related oral SCCs in India have a 5- to 10-fold DNA amplification of one or more of the c-*myc*, N-*myc*, K-*ras* and N-*ras* oncogenes, but no amplification of H-*ras* or L-*myc*. The question of *myc* and *ras* amplifications in the 57 cases presented in this study has also been investigated (unpublished data). A summary of this DNA amplification data is presented in Table II, indicating *ras* activation in Indian oral SCCs by both amplification as observed in K-*ras* and N-*ras*, and point mutations observed in H-*ras*.

The absence of either a normal H-*ras* codon 12 or 61 signal in 8/20 (40%) of the H-*ras* mutation positive samples on the dot blots is noteworthy. The inactivation or loss of normal H-*ras* alleles may be important in tumour progression, as demonstrated in colorectal (Baker *et al.*, 1989) and breast carcinoma (Theillet *et al.*, 1986). The apparent loss of the wild-type H-*ras* allele on chromosome 11, may be significant, not only for the loss of the H-*ras* gene itself, but for the loss or effective neutralisation of a nearby regulatory or tumour suppressor gene (Saxon *et al.*, 1986). Loss of H-*ras* allele has been reported in one of five oral SCC (in Canadian patients) by Howell and colleagues (Howell *et al.*, 1989).

A crucial question is at what stage of oral cancer development or progression do the H-*ras* mutations occur? It may be significant that seven of the 20 samples with H-*ras* mutations occur in stage II tumours, in contrast to *myc* and *ras* amplifications which generally accompany stage III and IV malignancies (Saranath *et al.*, 1989). Possibly H-*ras* mutations are important at an earlier stage of oral SCC development, with the *myc* and *ras* amplifications associated with the advanced stages (Saranath *et al.*, 1989). However, 12 of the 20 samples with activated *ras* contain a normal H-*ras* gene, and eight of these are $T_4$. Thus, loss of the normal H-*ras* allele in these tumours containing one mutated H-*ras* allele is not necessary for progression to the $T_4$ stage.

The oral cancer patients in this study were habitual tobacco chewers (5 to 15 years) and developed carcinomas at the site where the tobacco folded in 'quid' was kept for prolonged periods. Nitroso-containing compounds also present in tobacco, are known to induce H-*ras* mutations in experimental animals (Zarbl *et al.*, 1985; Quintanilla *et al.*, 1986). Our data strongly suggest that particular carcinogen(s), probably of nitroso origin, in tobacco, can affect specific base mutations at codons 12, 13 and 61 of H-*ras*, leading to oral malignancies. Different carcinogens present in tobacco, may be responsible for the two major types of mutations at H-*ras* 12.2 and 61.2, observed in our primary oral tumours. An equal number of transitions and transversions were found amongst our H-*ras* mutations. In general nitrosamines preferentially (but not exclusively) produce transitions whilst other carcinogens, like benzo(a)pyrene, cause transversions.

Preliminary studies of oral SCC from patients resident in the United Kingdom shows that H-*ras* mutations infrequently occur among such cancers (Chang *et al.*, unpublished). Mutations in codons 12, 13 and 61 of H-*ras*, K-*ras* and N-*ras* in these UK samples is a rare event. A possible reason for the difference in *ras* mutations frequency between UK and Indian tobacco-associated oral malignancies may be due to the mode of tobacco usage, the strains/species of tobacco used and the curing process in the two countries.

In summary, our data demonstrate that a major step by which chewing tobacco may cause oral malignancies is via carcinogen effected point mutations at codons 12, 13 and 61 of H-*ras*. This activation may occur at a relatively early stage in oral carcinogenesis. Further, inactivation or loss of the normal H-*ras* allele, may provide a selective advantage to the transformed clones. Since H-*ras* mutations may be biologically important at an early stage of oral carcinogenesis, such studies in premalignant lesions like leukoplakia are currently in progress in our laboratories. A correlation between the presence of H-*ras* mutations in premalignant lesions and malignant transformation, would further indicate the critical role of H-*ras* activation in oral carcinogenesis, and perhaps predisposition of these lesions towards oral malignancy.

The authors wish to thank Mrs Perin Notani for her advice in statistical analysis of the data and Mrs Manali Shelar and Ms C. Duckworth for typing the drafts and manuscript in Bombay and London. D. Saranath was supported in part by a grant from Imperial Cancer Research Fund, and S.E. Chang from Nuffield Foundation, London, UK.

### References

BAKER, S.J., FEARON, R.E., NIGRO, J.M. & 9 others (1989). Chromosome 17 deletions and P53 gene mutations in colorectal carcinomas. *Science*, **244**, 217.

BARBACID, M. (1987). *ras* genes. *Annu. Rev. Biochem.*, **56**, 779.

BINNIE, W.H. (1990). Low risk areas of the world. In *Risk Markers for Oral Diseases*. Vol. 2, Johnson, N.W. (ed.) Cambridge University Press: United Kingdom.

BINNIE, W.H. (1976). Epidemiology and etiology of oral cancer in Britain. *Proc. R. Soc. Med.*, **69**, 737.

BOS, J.L. (1989). *ras* oncogenes in human cancer: A Review. *Cancer Res.*, **49**, 4682.

BOS, J.L., FEARON, E.R., HAMILTON, S.R. & 4 others (1987). Prevalence of *ras* gene mutations in human colorectal cancers. *Nature*, **327**, 293.

BOS, J.L., TOKSOZ, D., MARSHALL, C.J. & 6 others (1985). Amino acid substitutions at codon 13 of the N-*ras* oncogene in human acute myeloid leukemia. *Nature*, **315**, 726.

DAFTARY, D.K. (1990). The situation in high risk areas of the world. In *Risk Markers for Oral Diseases*. Vol. 2, Johnson, N.W. (ed.). Cambridge University Press: United Kingdom.

FARR, C.J., MARSHALL, C.J., EASTY, D.J., WRIGHT, N.A., POWELL, S.C. & PARASKEVA, C. (1988). A study of *ras* gene mutations in colonic adenomas from familial polyposis coli patients. *Oncogene*, **3**, 673.

FIELD, J.K. & SPANDIDOS, D.A. (1987). Expression of oncogenes in human tumours with special reference to the head and neck region. *J. Oral Pathol.*, **16**, 97.

FORRESTOR, K., ALMOGUERA, C., HAN, K., GRIZZLE, W.E. & PERUCHO, M. (1987). Detection of high incidence of K-*ras* oncogenes during human carcinogenesis. *Nature*, **327**, 298.

FUJITA, J., SRIVASTAVA, S.K., KRAUS, M.H., RHIM, J.S., TRONICK, S.R. & AARONSON, S.A. (1985). Frequency of molecular alterations affecting *ras* protooncogenes in human urinary tract tumors. *Proc. Natl Acad. Sci. USA*, **82**, 3849.

GUPTA, P.C., MEHTA, F.S., PINDBORG, J.J., AGHI, M.B., BHONSLE, R.B. & MURTI, P.R. (1987). An educational intervention study for tobacco chewing and smoking habits among Indian villagers. In *Smoking and Health*, Hisamichi, A.M. & Tominaga, S. (eds) p. 623, Excerpta Medica: Amsterdam.

HOELLERING, J. & SHULER, C.F. (1988). Localization of H-*ras* mRNA in oral squamous cell carcinomas. *J. Oral Pathol. Med.*, **18**, 74.

HOWELL, R.E., WONG, F.S.H. & FENWICK, R.G. (1989). Loss of Harvey *ras* heterozygosity in oral squamous cell carcinoma. *J. Oral Pathol. Med.*, **18**, 79.

JANSSEN, J.W.G., STEENVOORDEN, A.C.M., LYONS, J. & 5 others (1987). *Ras* gene mutations in acute and chronic myelocytic leukemias, chronic myeloproliferative disorders and myelodysplastic syndromes. *Proc. Natl Acad. Sci USA*, **84**, 9228.

JUSSAWALLA, D.J. & DESHPANDE, V.A. (1971). Evaluation of cancer risk in tobacco chewers and smokers: an epidemiologic assessment. *Cancer*, **28**, 244.

KLEIN, G. & KLEIN, E. (1985). Evaluation of tumors and the impact of molecular oncology. *Nature*, **315**, 190.

LEMOINE, N.R., MAYALL, E.S., WYLLIE, F.W. & 4 others (1989). High frequency of *ras* oncogene activation in all stages of human thyroid tumorigenesis. *Oncogene*, **4**, 159.

MANIATIS, T., FRITSCH, E.F. & SAMBROOK, J. (1982). Isolation of high molecular weight eukaryotic DNA from cells grown in tissue culture. In *Molecular Cloning: A Laboratory Manual*, p. 280. Cold Spring Harbor Laboratory: New York, USA.

NEEDLEMAN, S.W., KRAUS, M.H., SRIVASTAVA, S.K., LEVINE, P.H. & AARONSON, S.A. (1986). High frequency of N-*ras* activation in acute myelogenous leukemia. *Blood*, **67**, 753.

PIERCE, J.H., EVA, A. & AARONSON, S.A. (1986). Interaction of oncogenes with haematopoietic cells. In *Clinics in Hematology: Acute Leukemia*. Gale, R.P. & Hoffbrand, A.V. (eds) Vol. 15, p. 573, Saunders: London.

PINDBORG, J.J. (1977). Epidemiological studies of oral cancer. *Intl. Dent. J.*, **27**, 172.

QUINTANILLA, M., BROWN, K., RAMSDEN, M. & BALMAIN, A. (1986). Carcinogen-specific mutation and amplification of Ha-*ras* during mouse skin carcinogenesis. *Nature*, **322**, 78.

SAIKI, R., SHARP, S., FALOONA, F. & 4 others (1985). Enzymatic amplification of $\beta$ globin genomic sequences and restriction site analysis for diagnosis of sickle cell anemia. *Science*, **230**, 1350.

SANGHAVI, L.D. (1981). Epidemiologic and intervention studies. Screening: cancer epidemiology: the Indian scene. *J. Cancer Res. Clin. Oncol.*, **9**, 1.

SARANATH, D., PANCHAL, R.G., NAIR, R., MEHTA, A.R., SANGHAVI, V.D. & DEO, M.G. (1990). Restriction fragment length polymorphism of the L-*myc* gene in oral cancer patients. *Br. J. Cancer*, **61**, 530.

SARANATH, D., PANCHAL, R.G., NAIR, R. & 5 others (1989). Oncogene amplification in squamous cell carcinoma of the oral cavity. *Jpn. J. Cancer Res.*, **80**, 430.

SAXON, P.J., SRIVASTAVA, E.S. & STANBRIDGE, E.J. (1986). Introduction of human chromosome 11 via microcell transfer controls tumorigenic expression of HeLa cells. *EMBO J.*, **5**, 3461.

TAPAROWSKY, E., SUARD, Y., FASANO, O., SHIMIZU, K. & GOLDFARB, M. (1982). Activation of the T24 bladder carcinoma transforming gene is linked to a single amino acid change. *Nature*, **300**, 762.

THEILLET, C., LIDERAU, R., ESCOT, C. & 5 others (1986). Loss of a c-H-*ras*-1 allele and aggressive human primary breast carcinomas. *Cancer Res.*, **46**, 4776.

UNION INTERNATIONALE CONTRE LE CANCER (1988). In *TNM Classification of Malignant Tumors*. Harmer, M.N. (ed.), Geneva.

VARMUS, H.E. (1984). The molecular genetics of cellular oncogenes. *Annu. Rev. Genet.*, **18**, 553.

WINN, D.M. (1984). Tobacco chewing and snuff dipping: an association with human cancer. In *N-Nitroso compounds: Occurrence, Biological Effects and Relevance to Human Cancer*. O'Neill, I.K., Von Borstel, R.C. & Miller, C.T. (eds), Geneva IARC Scientific Publ. No. 57, p. 837, IARC: Geneva.

WYNDER, E.L. & STELLMAN, S.D. (1977). Comparative epidemiology of tobacco related cancers. *Cancer Res.*, **37**, 4608.

YOKOTA, J., TSUNETSUGU-YOKOTA, Y., BATTIFORA, H., LEFEVRE, C. & CLINE, M.J. (1986). Alterations of myc, *myb* and *ras*-Ha proto-oncogene in cancers are frequent and show clinical correlation. *Science*, **231**, 261.

ZARBL, H., SUKUMAR, S., ARTHUR, A.V., MARTIN-ZANCA, D. & BARBACID, M. (1985). Direct mutagenesis of Ha-*ras*-1 oncogenes by N-nitroso-N-methylurea during initiation of mammary carcinogenesis in rats. *Nature*, **315**, 382.